# Efficacy of a Naturally Calcium and Magnesium-Rich Mineral Water on Musculoskeletal Fragility: A Randomized, Double-Blind Controlled Trial

**DOI:** 10.3390/nu18030470

**Published:** 2026-01-31

**Authors:** Antimo Moretti, Sara Liguori, Marco Paoletta, Francesca Gimigliano, Giovanni Iolascon

**Affiliations:** 1 Department of Medical and Surgical Specialties and Dentistry, University of Campania “Luigi Vanvitelli”, 80138 Naples, Italy; antimo.moretti@unicampania.it (A.M.); giovanni.iolascon@unicampania.it (G.I.); 2 Department of Mental and Physical Health and Preventive Medicine, University of Campania “Luigi Vanvitelli”, 80138 Naples, Italy; marco.paoletta@unicampania.it (M.P.); francesca.gimigliano@unicampania.it (F.G.)

**Keywords:** calcium, magnesium, mineral water, musculoskeletal fragility, osteosarcopenia, falls, muscle mass, randomized controlled trial

## Abstract

**Background/Objectives**: Calcium and magnesium play a key role in musculoskeletal health and neuromuscular function. Mineral waters naturally rich in these elements provide a highly bioavailable dietary source. This study evaluated whether daily intake of naturally calcium and magnesium-rich mineral water compared with low-mineral water affects fall risk, muscle mass, and muscle function in adults aged ≥50 years, with or without osteosarcopenia. **Methods**: In this 12-month, randomized, double-blind, controlled trial, 98 participants (86.7% women, 13.3% men) were assigned to consume 1 L/day of either SG9 (mineral water with high calcium and magnesium content) or J66 (low-mineral water). Outcomes included incidence of falls (primary endpoint), appendicular skeletal muscle mass (ASMM), muscle strength, physical performance, biochemical markers, and patient-reported measures. Assessments were performed at baseline, 6 months, and 12 months following CONSORT guidelines. **Results**: Ninety-eight participants (mean age ~63 years) were randomized and completed the 12-month follow-up. At 6 months, the incidence of falls was significantly lower in the SG9 group compared with the J66 group (relative risk reduction 82%; RR = 0.18, 95% CI 0.04–0.88; *p* < 0.05), whereas no significant between-group difference was observed at 12 months. Appendicular skeletal muscle mass indexed to height (ASMM/h^2^) was significantly higher in the SG9 group at 12 months (*p* = 0.0002). In participants with osteosarcopenia, SG9 intake was associated with a consistent improvement in ASMM/h^2^ at 6 and 12 months (*p* = 0.004 and *p* = 0.006, respectively). No significant between-group differences were detected in physical performance scores, biochemical markers, or quality-of-life measures. **Conclusions**: Daily consumption of calcium- and magnesium rich mineral water reduced fall risk and improved muscle mass in adults over 50 years, with or without osteosarcopenia. These findings support the role of calcium and magnesium-rich mineral water use as a complementary nutritional strategy for musculoskeletal health and fragility prevention.

## 1. Introduction

Calcium is an essential mineral involved in numerous physiological functions, including energy metabolism, muscle contraction, nerve impulse transmission, and bone mineralization [1]. Approximately 99% of the body’s calcium is stored in mineralized tissues, while the remaining fraction is distributed in body fluids and soft tissues, including muscle [2]. Serum calcium acts as the primary modulator of the calcium–vitamin D–parathyroid hormone (PTH) axis, tightly regulating PTH secretion through calcium-sensing receptors and, in turn, influencing vitamin D activation and systemic calcium homeostasis [3].

Magnesium also plays a key role in musculoskeletal physiology, serving as a cofactor in more than 300 enzymatic reactions, including ATP synthesis, muscle contraction and relaxation, neuromuscular transmission, and vitamin D metabolism [4]. Suboptimal magnesium intake is common in older adults and has been associated with reduced muscle performance, impaired bone quality, and increased fall risk [5]. Importantly, magnesium and calcium share interconnected regulatory pathways, and inadequate magnesium status may compromise calcium homeostasis and overall musculoskeletal function [6].

Calcium deficiency is prevalent among older adults and represents a major risk factor for osteoporosis, sarcopenia, and falls, thereby increasing the likelihood of fragility fractures [7]. Sarcopenia is characterized by the progressive loss of muscle mass and strength, and is frequently associated with aging [8]. Its coexistence with osteoporosis has led to the definition of osteosarcopenia, a multifactorial condition with substantial healthcare and social costs. Among the modifiable risk factors, nutrition plays a central role. Adequate intake of calcium has been associated with higher bone mineral density (BMD) and improved muscle function, including fall risk reduction [9,10,11].

Traditionally, dairy products are the main dietary sources of calcium. However, their consumption may be limited by lactose intolerance, dietary preferences, or medical contraindications [12]. In this context, mineral waters naturally rich in calcium and magnesium represent a valid alternative, as the bioavailability of calcium from these waters is comparable to that from dairy sources, while simultaneously providing a readily absorbable source of magnesium [13,14]. This aspect is particularly relevant for postmenopausal women, for whom sufficient intake of both minerals is crucial for preventing bone loss and supporting neuromuscular function. While the beneficial effects of calcium-rich water on bone health are well recognized, its impact on muscle mass and function remains less clearly defined.

Several epidemiological and clinical studies have demonstrated an association between higher calcium intake—including from drinking water—and improved bone health. As early as 1979, Matković et al. [15] studied two Yugoslavian populations living in areas with differing calcium concentrations in drinking water. Their findings showed that higher calcium intake was associated with increased cortical bone mass and a lower incidence of fractures. Later, Cepollaro et al. [16] confirmed that, in early postmenopausal women, regular consumption of calcium-rich mineral water can help maintain BMD. Despite this growing body of evidence, data on effects of mineral waters consumption on muscle mass and function remain limited. Given the high bioavailability of calcium contained in naturally calcium-rich mineral waters, and the synergistic role of magnesium in neuromuscular function, the daily intake of 1 L of calcium and magnesium-rich mineral water may help maintain musculoskeletal function, support the preservation of muscle mass, and potentially slow age-related muscle loss, particularly in individuals with musculoskeletal fragility. However, clinical evidence on the effects of such waters on muscle mass and fall risk remains limited, underscoring the need for controlled trials to clarify these potential benefits.

The primary aim of this study is to evaluate the efficacy of daily intake of naturally calcium and magnesium-rich mineral water on fall risk, muscle mass and function, in adults over the age of 50, with or without musculoskeletal fragility.

## 2. Materials and Methods

### 2.1. Study Design

This study is a 12-month, randomized, double-blind, controlled clinical trial, conducted at the Unit of Physical and Rehabilitation Medicine of the University Hospital “Luigi Vanvitelli” in Naples, Italy. The protocol was developed in accordance with the principles of the Declaration of Helsinki, approved by the local Ethics Committee (Protocol No. 0035084/i, dated 15 December 2023), and registered on ClinicalTrials.gov (Identifier: NCT06851676). The trial was reported following the CONSORT guidelines for randomized controlled trials [17].

### 2.2. Participants

Adults aged ≥50 years, community-dwelling and ambulatory, referring to the rehabilitation outpatient service, were eligible for inclusion. Participants could present with normal musculoskeletal status or with osteopenia, osteoporosis, sarcopenia, or osteosarcopenia, diagnosed either previously or at baseline according to standardized criteria (DXA-based T-scores for bone status and European Working Group on Sarcopenia in Older People 2—EWGSOP2-criteria for sarcopenia). All participants were required to be independent in basic activities of daily living and not receive pharmacological calcium and/or magnesium supplementation. Participants were consecutively screened among all adults aged ≥50 years attending the outpatient rehabilitation service during the recruitment period. Screening was performed systematically by trained investigators according to predefined eligibility criteria. This recruitment strategy was designed to reflect a real-world clinical population at risk of musculoskeletal fragility, thereby enhancing the external validity of the findings. We excluded individuals with secondary causes of osteoporosis, such as prolonged glucocorticoid therapy, chronic liver or kidney disease, endocrine disorders, or malignancies, or musculoskeletal impairments leading to loss of independence in activities of daily living (ADLs). Other exclusion criteria included neurodegenerative or inflammatory conditions, advanced degenerative joint diseases, chronic obstructive pulmonary disease, psychiatric disorders that could impair informed consent, and pregnancy or lactation. All participants provided written informed consent before enrollment.

### 2.3. Intervention

The intervention consisted of daily consumption of one liter of mineral water. Participants were randomized to receive 1 L/day of Fonte Fausta^®^ (Ferrarelle Società Benefit, Riardo, Italy, SG9 code, naturally calcium and magnesium-rich mineral water; calcium 587 mg/L, magnesium 128 mg/L, sodium 18.8 mg/L, bicarbonate 256 mg/L) or Natia^®^ (Ferrarelle Società Benefit, Riardo, Italy, J66 code, low-mineral water; calcium 36 mg/L, magnesium 4.9 mg/L, sodium 30 mg/L, bicarbonate 230 mg/L). Participants were instructed to consume 1 L/day of the assigned water, distributed throughout the day. Both waters were still and packaged identically to ensure blinding. Randomization was performed using computer-generated blocks, stratified by sex and osteosarcopenia status, with allocation concealed in sealed envelopes held by Ferrarelle S.p.A., Riardo, Caserta. Allocation concealment was guaranteed by sealed coding, which was released only after completion of data collection and statistical analysis. Neither participants nor investigators were aware of group assignments throughout the study, ensuring a double-blind design.

### 2.4. Outcome

The primary outcome of the trial was the incidence of falls, assessed at six and twelve months, considered as a key outcome of musculoskeletal fragility. Alongside this, a broad set of secondary outcomes was evaluated to capture structural, functional, biochemical, and patient-reported dimensions of musculoskeletal health. Body composition was assessed by bioelectrical impedance analysis (BIA 101 BIVA^®^ PRO, Akern, Pisa, Italy) to measure appendicular skeletal muscle mass (ASMM), while muscle strength was measured using handgrip dynamometry (Jamar dynamometer, Sammons Preston Rolyan, Bolingbrook, IL, USA). Comorbidity burden was assessed using the Cumulative Illness Rating Scale (CIRS), which evaluates the severity and number of chronic medical conditions across multiple organ systems. The CIRS Severity Index, Comorbidity Index, and Total Score were calculated and used to characterize baseline clinical complexity and comparability between groups. Physical performance was assessed using the Short Physical Performance Battery, including balance, gait speed, and sit-to-stand testing, along with a direct measurement of gait speed in meters per second. The level of physical activity was determined using the International Physical Activity Questionnaire (IPAQ) and objectively measured in metabolic equivalent of task (MET) units using the ActiGraph GT3X+ accelerometer. Biochemical outcomes comprised serum calcium, phosphate, magnesium, creatinine, parathyroid hormone (PTH), 25-hydroxyvitamin D, alkaline phosphatase (ALP), bone-specific ALP, and inflammatory markers such as C-reactive protein (CRP) and erythrocyte sedimentation rate (ESR). In addition, bone turnover markers, myokines, and adipokines were analyzed, including CTX, P1NP, P3NP, sclerostin, irisin, leptin, and TNF-α. Urinary outcomes were also collected, consisting of 24 h calcium, magnesium, and phosphate excretion, together with creatinine clearance. Finally, patient-reported outcomes were assessed using the EuroQoL-5D-3L index and visual analog scale, complemented by a 7-point Likert scale evaluating the acceptability of the intervention. All assessments were performed at baseline (T0), 6 months (T1), and 12 months (T2), under standardized conditions (morning, fasting, same operator).

### 2.5. Statistical Analysis

Sample size was calculated a priori to detect a 20% relative reduction in fall incidence with 80% power and a two-sided α of 0.05, resulting in a target enrollment of 98 participants (49 per group). All statistical analyses were performed using IBM SPSS Statistics, version 29 (IBM Corp., Armonk, NY, USA). Continuous variables were expressed as mean ± standard deviation (SD) or median and interquartile range (IQR), according to their distribution, assessed using the Shapiro–Wilk test. Categorical variables were reported as absolute frequencies and percentages. Baseline characteristics were compared between groups according to variable type: parametric or non-parametric tests for continuous and ordinal variables, and χ^2^ or Fisher’s exact test for categorical variables. For each group, comparisons across the time points were conducted using the Friedman test. When the overall test was significant, post hoc pairwise comparisons between time points were carried out using the Wilcoxon test. For between-group comparisons at the time points, a multivariate General Linear Model (GLM) was applied to evaluate mean differences across groups. Bonferroni-adjusted post h tests were used to control multiple comparisons. In addition, fall risk at 6 and 12 months was compared between groups at each time point separately using the χ^2^ test. Relative risk (RR) and 95% confidence intervals were calculated to quantify between-group differences. A two-tailed *p*-value < 0.05 was considered statistically significant. The sponsor had no role in data analysis or interpretation and did not have access to the study data. All analyses were conducted independently by the investigators.

## 3. Results

**Figure 1:**
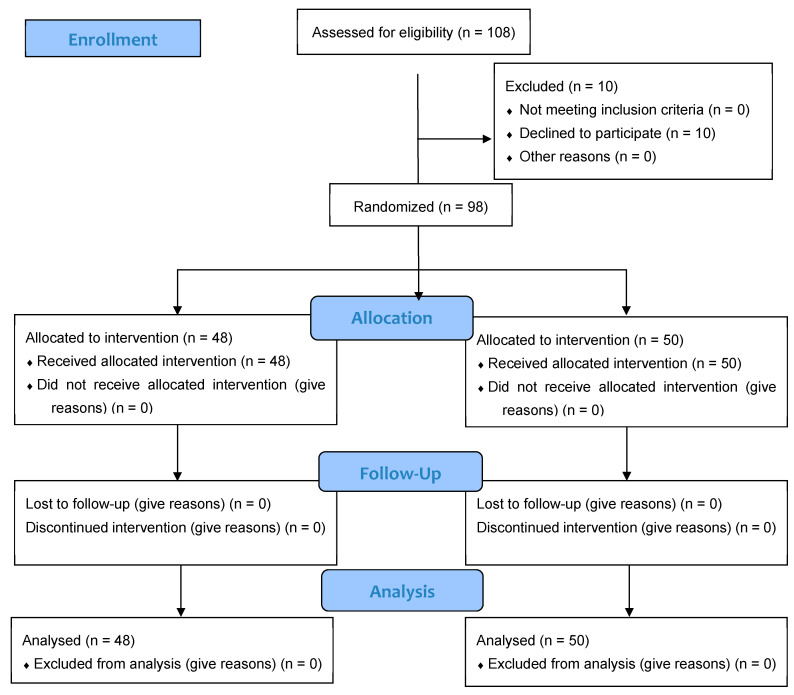
CONSORT flow diagram illustrating the progress of participants through the phases of the randomized, double-blind controlled trial, including enrollment, allocation, follow-up, and analysis.

**Figure 2:**
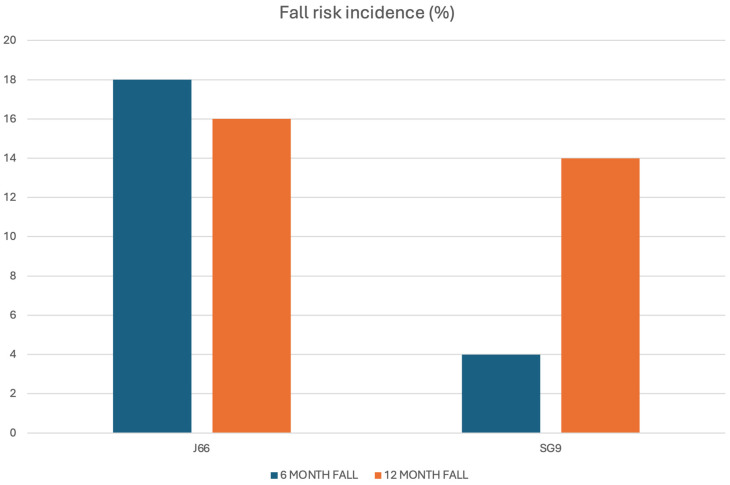
Six- and twelve-month fall incidence in the J66 and SG9 groups.

A total of 108 individuals were screened for eligibility, of whom 98 (86.7% women, 13.3% men) met inclusion criteria and were randomized to receive either J66 water (*n* = 48) or SG9 water (*n* = 50). The osteosarcopenic subgroup included 20 patients (93.3% women, 6.7% men; J66 water *n* = 8 and SG9 water *n* = 12). All participants completed the 12-month follow-up, with no losses or discontinuations (Figure 1). Baseline demographic, biochemical, and functional characteristics were generally comparable between groups; no significant baseline differences were observed within the osteosarcopenic subgroup (Table 1). Fall risk differed significantly between groups. At the 6-month fall risk assessment, conducted using the χ^2^ test, a comparison between the two groups revealed that 9 patients in the J66 group and 2 patients in the SG9 group had experienced at least one fall in the previous six months (“fallers”). The risk of falling in the SG9 group was 82.0% lower than the J66 group (RR = 0.18; 95% CI: 0.04–0.88). In the 12-month analysis (T2), the risk of falling was not statistically significant between water groups (RR = 0.69; 95% CI: 0.227–2.129) (Figure 2).

In the osteosarcopenic subgroup, both 6- and 12-month risk of fall was not statistically significant (T1 *p* = 0.069; T2 *p* = 0.536).

In general population, at 6 months, no significant differences were observed between the two groups in serum and urinary biochemical markers or patient-reported outcomes, except for urinary calcium and ASMM, that were significantly higher in SG9 group (Table 2). Measures of physical performance, including SPPB total score, balance, gait speed, sit-to-stand, and handgrip strength, did not differ significantly between groups.

**Figure 3:**
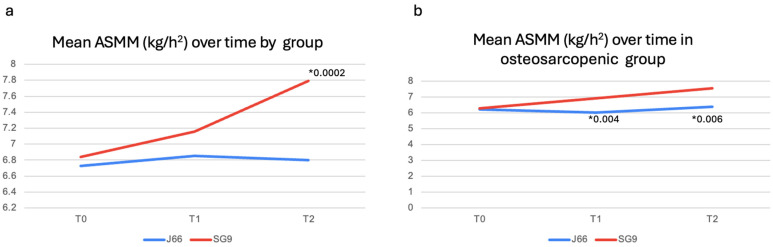
Muscle mass changes in general population (**a**) and in osteosarcopenic patients (**b**). Abbreviations: Appendicular Skeletal Muscle Mass (ASMM). * *p*-value.

After 12 months, serum and urinary biochemical parameters remained largely comparable between groups (Table 3). However, urinary magnesium excretion was significantly higher in the SG9 group (*p* = 0.009), while urinary calcium excretion was significantly lower in the J66 group (*p* = 0.006). Functional outcomes, including SPPB components, gait speed, and handgrip strength, did not differ significantly between groups at 12 months. ASMM/h^2^ was significantly higher in the SG9 group compared with the J66 group at 12 months (mean difference = −0.99 kg/m^2^; 95% CI: 0.485 to 1.495; *p* = 0.0002) (Figure 3a). A predefined subgroup analysis was conducted in participants with osteosarcopenia (J66: *n* = 8; SG9: *n* = 12). At 6 months, no significant differences were detected between groups for biochemical markers or functional outcomes, except for a significant increase in handgrip strength in the SG9 group (*p* = 0.04) (Table 2). At 12 months, urinary calcium excretion was significantly higher in the SG9 group (*p* = 0.018). At both 6- and 12- months, subgroup analyses highlighted a significant improvement in ASMM/h^2^ in participants receiving SG9, with values higher than those observed in the J66 group (T1: mean difference = 0.88 kg/m^2^; 95% CI: 0.37 to 1.41; *p* = 0.004 T2: mean difference = 1.16 kg/m^2^; 95% CI: 0.455; 1.865; *p* = 0.006, respectively) (Table 3) (Figure 3b).

**Figure 4:**
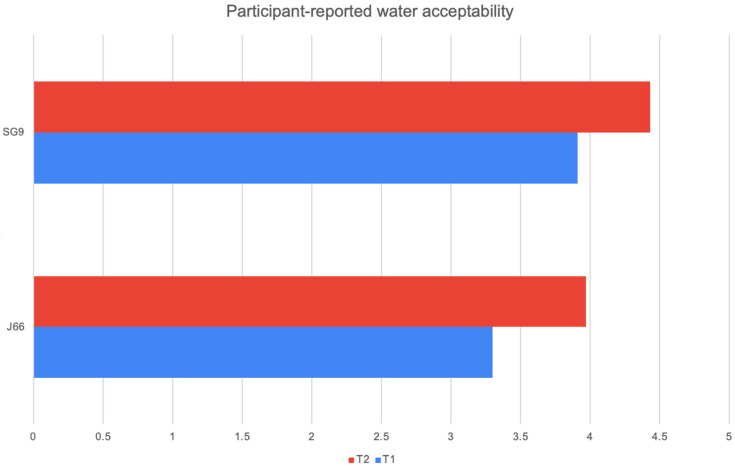
Participant-reported water acceptability.

At both 6 and 12 months, participants’ ratings of water palatability showed a consistent preference for the SG9 water compared with J66. In the J66 group, mean acceptability increased from 3.30 at the first assessment to 3.97 at 12 months, indicating gradual improvement over time. In contrast, the SG9 group started with a higher baseline appreciation (3.91) and further increased to 4.43 at 12 months. This pattern suggests that SG9 water was not only better accepted from the outset but also continued to gain favor throughout the follow-up period. However, despite these numerical differences, none of the comparisons reached statistical significance, indicating that the observed trends should be interpreted with caution (J66 *p*-value = 0.746 SG9 *p*-value = 0.064) (Figure 4).

## 4. Discussion

**Figure 5:**
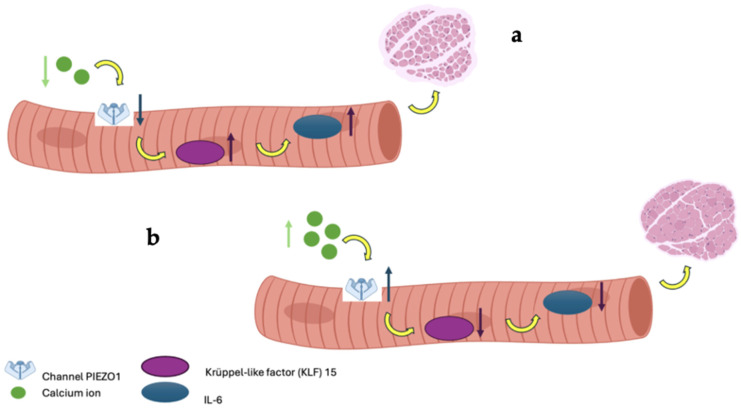
In image (**a**), low calcium bioavailability leads to a reduction in PIEZO1 channel expression, resulting in the upregulation of the genes KLF-15 and IL-6, which induce muscle atrophy; conversely, in image (**b**), adequate calcium bioavailability maintains PIEZO1 expression, promoting healthy muscle mass.

This randomized, double-blind controlled trial investigated the efficacy of a naturally calcium and magnesium-rich mineral water on fall risk reduction, muscle mass, and muscle strength improvements in patients with or without a diagnosis of osteosarcopenia. The main findings indicate a transient reduction in fall incidence at 6 months and a significant increase in ASMM/h^2^ at 12 months, particularly among participants with osteosarcopenia. However, no consistent changes were observed in muscle strength, physical performance, or other functional outcomes, highlighting the need for cautious interpretation of these results. The reduction in fall incidence observed at 6 months in the intervention group was not sustained at 12 months. This temporal pattern likely reflects the multifactorial and dynamic nature of falls in older adults. Short-term improvements may reflect early neuromuscular benefits of optimized calcium and magnesium availability, potentially influencing muscle excitability and postural control before measurable changes in strength or performance occur. Over time, however, fall risk is increasingly influenced by behavioral, environmental, and clinical factors that are not directly modulated by mineral intake alone [18]. Moreover, the relatively low number of fall events at 12 months and the wide confidence intervals may have limited the statistical power to detect persistent between-group differences. Despite a significant increase in appendicular skeletal muscle mass at 12 months, most notably in the osteosarcopenic subgroup, no parallel improvements in muscle strength or physical performance were detected, supporting the concept that gains in muscle mass per se may be insufficient to ensure sustained fall prevention, particularly when assessed over a relatively short time frame and in the absence of structured exercise interventions. Taken together, these findings suggest that calcium and magnesium-rich mineral water may contribute to early fall risk reduction and long-term preservation of muscle mass, but that durable fall prevention likely requires a multimodal approach integrating nutritional strategies with targeted exercise and balance interventions. The dual high content of calcium and magnesium in SG9 may contribute synergistically to neuromuscular function, potentially explaining the observed reduction in falls and the improvement in muscle mass. Magnesium plays a key role in ATP production, muscle contraction and relaxation, neuromuscular transmission, and vitamin D activation; thus, its co-delivery with calcium may enhance the physiological impact on muscle performance and balance [4]. These findings align with the growing body of evidence supporting the importance of adequate mineral intake, particularly calcium and magnesium, in fall prevention. A recent Japanese observational study involving over 38,000 adults reported that low dietary calcium intake was significantly associated with an increased risk of falls, with adjusted odds ratios of 1.29 in men and 1.12 in women (Lowest Vs. Highest quartile) in cross-sectional analyses, with similar results confirmed longitudinally after a 5-year follow-up [19]. Although the study by Asano et al. assessed calcium intake from food rather than water, its findings reinforce the hypothesis that sufficient calcium intake, regardless of source, may help maintain musculoskeletal efficiency and reduce the risk of falls and, ultimately, fragility fractures. Magnesium intake has also been associated with improved muscle performance, reduced frailty, and better physical function in older adults, further supporting the biological plausibility of our results [5,20,21]. Our study is among the few to specifically evaluate the impact of mineral water intake providing both calcium and magnesium on musculoskeletal health. Previous research has shown that the bioavailability of calcium from mineral waters is comparable to, or even greater than, that from milk and dairy products, supporting the hypothesis that such waters may represent an effective and accessible source of dietary calcium, especially for individuals who are lactose intolerant or have low adherence to dairy consumption [1]. Importantly, magnesium from mineral water is also highly bioavailable, and its co-absorption may facilitate optimal calcium utilization and endocrine regulation [22]. The intestinal absorption of calcium from certain mineral waters can reach levels similar to or higher than those of traditional calcium sources, making them a useful alternative for maintaining musculoskeletal health [1]. Recent evidence indicates that calcium is not only essential for bone health and muscle contraction but may also influence muscle mass through various cellular mechanisms [23]. Specifically, intracellular calcium contributes to muscle mass maintenance Via the activity of the mechanosensitive channel Piezo1. Reduced Piezo1 expression, as seen during immobilization, is associated with activation of the KLF15/IL-6 pathway and muscle atrophy. Furthermore, calcium may indirectly regulate protein synthesis through the AMPK/mTOR signaling pathway. Magnesium, in turn, is required for ATP stability, ribosomal function, and protein synthesis, and low magnesium status has been linked to reduced muscle mass and strength. Together, these mechanisms support the hypothesis that adequate calcium and magnesium supply, including through mineral water intake, may help maintain muscle mass, especially in individuals with osteosarcopenia (Figure 5) [24]. The physiological increase in urinary calcium observed in the SG9 group, remaining within normal limits, likely reflects improved calcium bioavailability and a more efficient calcium–vitamin D–PTH axis, with reduced parathyroid stimulation. Similarly, the increase in urinary magnesium observed at 12 months is consistent with the higher magnesium content of SG9 and may indicate adequate absorption and renal handling of the mineral. This optimized mineral homeostasis may represent a key mechanistic link between adequate calcium and magnesium intake and the observed reduction in fall risk and improvement in muscle mass, particularly in individuals with osteosarcopenia [25]. In this context, urinary calcium and magnesium emerge as integrative and clinically meaningful indicators of effective mineral utilization at the musculoskeletal level, reinforcing the concept that functional outcomes may depend more on mineral bioavailability and endocrine balance than on static serum measurements alone. These biochemical findings support adequate mineral homeostasis and bioavailability but should be interpreted cautiously, as they do not directly demonstrate causal effects on musculoskeletal outcomes. In our study, daily intake of SG9 resulted in a significant increase in ASMM, suggesting a potential role in supporting the maintenance of muscle mass and slowing its age-related decline. The reduction in fall risk observed at 6 months further indicates a possible benefit on overall musculoskeletal function, consistent with the physiological roles of calcium and magnesium in muscle contraction, neuromuscular transmission, and postural stability. The high assimilability of calcium from mineral waters such as SG9, well documented in the previous literature, is supported by the stable biochemical profile observed in our cohort and by the increased urinary magnesium excretion in the intervention group. Although our study did not directly assess bone mineral density or fracture incidence, the reduction in falls represents a clinically meaningful outcome in the prevention of fragility fractures, particularly in individuals with osteopenia or osteoporosis. This study has several strengths. To our knowledge, it represents the first randomized, double-blind controlled trial specifically investigating the efficacy of a naturally calcium and magnesium rich mineral water on both functional and metabolic outcomes related to musculoskeletal health. The pragmatic trial design, including adults over 50 years at increased risk of musculoskeletal fragility and minimal interference with usual lifestyle habits, enhances real-world applicability. The long follow-up period, high adherence, and blinding procedures strengthen internal validity. Moreover, the integrated evaluation of falls, physical performance, body composition, biochemical markers, and patient-reported outcomes provides a comprehensive and clinically meaningful assessment that aligns with current multidimensional approaches to musculoskeletal health. However, several limitations of the present study should be acknowledged. First, although the trial was adequately powered for the primary outcome, it was not powered to detect definitive effects within specific clinical subgroups. Participants included adults over 50 years with heterogeneous musculoskeletal profiles, ranging from individuals without diagnosed musculoskeletal fragility to those with osteosarcopenia. Consequently, all subgroup analyses should be considered exploratory and hypothesis-generating rather than confirmatory, particularly for the osteosarcopenic subgroup, which included a limited number of participants. Second, the heterogeneity of the enrolled population, while increasing the external validity and pragmatic relevance of the study, may have diluted intervention effects in the overall analysis and complicated the interpretation of subgroup-specific findings. Future studies specifically designed and powered for homogeneous populations are needed to confirm these observations. Third, the study population was predominantly female, reflecting the epidemiology of osteosarcopenia and related conditions in older adults. However, this sex imbalance limits the generalizability of the findings to male populations and precluded sex-stratified analyses. Fourth, although participants were instructed to maintain their usual diet throughout the study period, detailed dietary intake was not strictly controlled. Therefore, residual confounding from unmeasured nutritional or behavioral factors cannot be completely excluded. Moreover, the dietary intake of calcium and magnesium from food sources was not formally assessed or controlled. Although participants were required not to receive pharmacological supplementation, variability in habitual dietary intake of these minerals cannot be excluded and may have influenced the results. Finally, muscle mass was assessed using BIA, which provides indirect estimates of body composition and is inherently less precise than imaging-based reference techniques such as DXA. However, according to EWGSOP 2 criteria for sarcopenia, BIA is an acceptable, portable method to measure body composition, to determine low muscle quantity, though hydration can affect result Therefore, the observed changes in muscle mass should be interpreted with caution and considered indicative rather than definitive evidence of true changes in skeletal muscle mass [26,27]. While the study followed a double-blind randomized design and included an extensive panel of biochemical and functional outcomes, the mechanistic pathways underlying the observed effects of calcium and magnesium-rich mineral water on muscle and bone metabolism cannot be fully elucidated within the present framework and warrant further investigation in targeted clinical studies. Overall, our findings suggest that daily consumption of mineral water naturally rich in calcium and magnesium may contribute to the preservation of muscle mass and to a transient reduction in fall incidence, particularly in individuals with osteosarcopenia. However, these effects were not accompanied by consistent improvements in muscle strength or physical performance and should therefore be interpreted cautiously.

## 5. Conclusions

The daily intake of 1 L of SG9, a mineral water rich in bioavailable calcium and magnesium, was associated with a transient reduction in fall incidence at 6 months and with an increase in ASMM/h^2^ at 12 months, particularly in participants with osteosarcopenia. However, these changes were not accompanied by consistent improvements in muscle strength, physical performance, or other functional outcomes. Taken together, these findings suggest that calcium and magnesium-rich mineral water may contribute to the preservation of muscle mass and to short-term improvements in fall-related outcomes, especially in individuals with musculoskeletal fragility, but do not support broad conclusions regarding overall musculoskeletal health. Further adequately powered, long-term studies—ideally incorporating imaging-based muscle assessment techniques and combined nutritional and exercise interventions—are needed to confirm these findings, clarify their clinical relevance, and determine potential implications for bone health and fracture prevention.

## Figures and Tables

**Table 1:** Baseline comparison of general characteristics, laboratory parameters, and functional scales between the two water groups, in the general population and in the osteosarcopenic subgroup.

	General Population			Osteosarcopenic Subgroup		
Parameters	J66 Water (N = 48)	SG9 Water (N = 50)	*p*-Value	J66 Water (N = 8)	SG9 Water (N = 12)	*p*-Value
Sex *n* (%)	43 women (89.6%) 5 men (10.4%)	42 women (84%) 8 men (16%)	0.410	8 women (100%)	9 women (75%) 3 men (25%)	0.230
Age (years)	64.08 ± 6.61	62.96 ± 8.10	0.424	68.42 ± 7.11	65.5 ± 8.53	0.424
BMI (kg/m^2^)	24.52 ± 3.71	26.23 ± 4.69	0.065	22.15 ± 2.57	25.54 ± 3.94	0.076
Creatinine (mg/dL)	0.77 ± 0.16	0.77 ± 0.12	0.927	0.73 ± 0.15	0.77 ± 0.06	0.463
ALT (U/L)	18.40 ± 7.80	19.38 ± 8.34	0.456	18.29 ± 7.65	25.50 ± 15.52	0.462
AST (U/L)	21.42 ± 4.65	21.76 ± 6.32	0.628	20.86 ± 3.67	26.88 ± 11.37	0.536
Magnesium (mEq/L)	2.01 ± 0.17	2.03 ± 0.24	0.896	2.08 ± 0.06	2.06 ± 0.15	0.776
Phosphate (mg/dL)	3.45 ± 0.47	3.40 ± 0.43	0.559	3.47 ± 0.36	3.35 ± 0.46	0.561
Calcium (mg/dL)	9.45 ± 0.45	9.50 ± 0.44	0.889	9.5 ± 0.16	9.29 ± 0.45	0.557
Total ALP (U/L)	72.35 ± 23.83	75.08 ± 18.96	0.463	71.57 ± 19.75	84.63 ± 21.05	0.247
25OH-Vitamin D (ng/mL)	32.02 ± 10.15	31.87 ± 10.82	0.907	38.47 ± 8.55	32.41 ± 12.48	0.280
bALP (%)	11.85 ± 4.94	12.80 ± 8.64	0.710	12.45 ± 4.54	14.22 ± 6.41	0.537
PTH (pg/mL)	33.66 ± 11.99	34.56 ± 15.11	0.930	35.15 ± 19.96	35.90 ± 18.13	0.939
Magnesium (urinary, mEq/24 h)	8.05 ± 3.66	8.36 ± 4.63	0.970	5.92 ± 1.53	6.72 ± 3.67	0.821
Phosphate (urinary, mg/24 h)	596.81 ± 248.28	645.62 ± 323.46	0.409	439.71 ± 110.8	483.88 ± 323.46	0.353
Calcium (urinary, mg/24 h)	164.37 ± 95.99	165.86 ± 116.43	0.802	142.42 ± 72.65	113.05 ± 100.17	0.554
Creatinine clearance (mL/min)	87.05 ± 25.98	95.56 ± 52.22	0.947	63.14 ± 17.3	115.00 ± 101.88	0.185
TNF-Alfa (pg/mL)	44.88 ± 70.17	44.06 ± 124.5	0.265	3.42 ± 5.19	25.14 ± 60.33	0.486
P3NP (μg/L)	10.59 ± 3.30	10.31 ± 2.72	0.594	10.42 ± 2.82	8.52 ± 2.54	0.758
Sclerostin (pmol/L)	74.21 ± 50.53	67.05 ± 45.99	0.518	48.1 ± 25.02	54.53 ± 39.29	0.787
Leptin (ng/mL)	12.25 ± 11.00	17.00 ± 18.62	0.593	8.04 ± 5.6	14.11 ± 12.89	0.355
CTX (ng/mL)	0.21 ± 0.17	0.24 ± 0.29	0.657	0.23 ± 0.18	0.41 ± 0.62	0.728
IRISIN (ng/mL)	140.83 ± 38.5	137.99 ± 39.89	0.341	134.47 ± 40.27	111.06 ± 59.19	0.616
P1NP (ng/mL)	544.36 ± 258.24	503.31 ± 235.29	0.334	481.24 ± 148.73	524.15 ± 311.05	0.817
CIRS Severity Index	1.37 ± 0.24	1.33 ± 0.24	0.226	1.55 ± 0.29	1.39 ± 0.31	0.096
CIRS Comorbidity Index	2.0 (0–6)	1.0 (0–6)	0.601	4.0 (0–6)	2.50 (0–6)	0.256
CIRS Total Score	5 (0–13)	4 (0–13)	0.110	7 (1–13)	5 (0–13)	0.151
EuroQoL-5D Index	0.99 ± 0.09	0.97 ± 0.10	0.110	0.96 ± 0.09	1.01 ± 0.03	0.610
EuroQoL-5D (VAS)	70.52 ± 13.50	72.80 ± 13.75	0.527	73.57 ± 13.75	68.75 ± 13.56	0.430
SPPB Total Score	7.0 (1–11)	7.0 (2–12)	0.173	5.0 (1–8)	5.50 (2–8)	0.876
SPPB Balance	3.0 (0–4)	4.0 (0–4)	0.155	2.0 (0–4)	1.0 (0–4)	1.000
SPPB Gait Speed	2.0 (1–4)	2.0 (1–4)	0.178	1.0 (1–2)	2.0 (1–2)	0.276
SPPB Sit-to-Stand	2.0 (0–4)	2.0 (0–4)	0.470	2.0 (0–3)	1.50 (0–3)	0.904
Handgrip Strength (kg)	22.98 ± 6.86	23.69 ± 8.64	0.577	15.48 ± 0.68	15.61 ± 2.74	0.876
ASMM (kg)	17.68 ± 3.13	19.19 ± 4.74	0.063	15.57 ± 1.99	18.03 ± 3.51	0.063
ASMM (kg)/h^2^ (m)	6.73 ± 0.92	6.84 ± 1.71	0.055	6.24 ± 0.57	6.31 ± 2.0	0.057
IPAQ (MET)	4083.02 ± 3989.10	4717.61 ± 3821.65	0.346	2962.14 ± 1539.72	1360.63 ± 800.84	0.589
MET (total)	11,817.29 ± 3572.44	11,735.26 ± 4169.68	0.744	9528.00 ± 4805.66	8033.88 ± 4294.99	0.758
MET (week)	1771.20 ± 517.54	1765.60 ± 531.18	0.755	1544.57 ± 720.09	1677.63 ± 296.44	0.396

Notes: Continuous variables are expressed as mean ± standard deviation or median with interquartile range; categorical variables are expressed as total number (%). All pairwise time point comparisons were significant. Abbreviations: Body Mass Index (BMI), Alanine Aminotransferase (ALT), Aspartate Aminotransferase (AST), Total Alkaline Phosphatase (Total ALP), 25-Hydroxy Vitamin D (25OH-Vitamin D), Bone-specific Alkaline Phosphatase (bALP), Parathyroid Hormone (PTH), Tumor Necrosis Factor-alpha (TNF α), Procollagen Type III N-terminal Propeptide (P3NP), C-terminal Telopeptide of Type I Collagen (CTX), Procollagen Type I N-terminal Propeptide (P1NP) (µg/L), Cumulative Illness Rating Scale (CIRS), EuroQoL-5Dimension (EuroQoL-5D), Visual Analog Scale (VAS), Short Physical Performance Battery (SPPB), Handgrip Strength Examination (HGSE), Appendicular Skeletal Muscle Mass (ASMM), International Physical Activity Questionnaire (IPAQ); Metabolic Equivalent of Task (MET).

**Table 2:** Between-group comparison after 6 months of laboratory parameters, and functional outcomes in the general population and osteosarcopenic subgroup, divided by type of water.

	General Population					Osteosarcopenic Population				
Dependent Variable	J66 N = 48 (M + SD)	SG9 N = 50 (M + SD)	Mean Difference	95% CI	*p*-Value	J66 N = 8 (M + SD)	SG9 N = 12 (M + SD)	Mean Difference	95% CI	*p*-Value
BMI	24.99 ± 4.44	25.68 ± 3.82	0.69	[−0.97, 2.35]	0.41	24.39 ± 2.80	24.86 ± 4.82	0.47	[−3.57, 4.51]	0.79
Magnesium (mEq/L)	2.064 ± 0.183	2.242 ± 0.837	−0.02	[−0.093, 0.049]	0.541	2.06 ± 0.09	2.06 ± 0.17	0.00	[−0.14, 0.14]	1.000
Phosphate (mg/dL)	3.612 ± 0.448	3.604 ± 0.481	−0.01	[−0.110, 0.084]	0.787	3.47 ± 0.12	3.80 ± 0.81	0.33	[−0.23, 0.89]	0.569
Calcium (mg/dL)	9.455 ± 0.381	9.100 ± 1.987	−0.35	[−0.923, 0.213]	0.21	9.33 ± 0.06	9.36 ± 0.34	0.03	[−0.21, 0.27]	0.77
Total ALP (U/L)	76.94 ± 25.98	75.04 ± 17.26	−1.90	[−10.77, 6.97]	0.67	86.00 ± 31.77	83.40 ± 22.72	−2.60	[−33.35, 28.15]	0.85
25OH-Vitamin D (ng/mL)	28.64 ± 9.99	26.64 ± 9.48	−2.00	[−5.91, 1.91]	0.31	29.50 ± 4.33	27.98 ± 13.17	−1.52	[−11.22, 8.18]	0.72
bALP (%)	10.46 ± 4.42	9.83 ± 3.60	−0.63	[−2.25, 0.99]	0.44	14.50 ± 5.76	10.64 ± 4.59	−3.86	[−9.61, 1.89]	0.16
PTH (pg/mL)	36.88 ± 13.68	34.05 ± 12.83	−2.83	[−8.15, 2.49]	0.29	28.43 ± 8.43	28.30 ± 9.38	−0.13	[−3.71, 3.44]	0.94
Urinary Magnesium (mEq/24 h)	11.05 ± 4.22	10.76 ± 3.33	0.29	[−1.82, 1.24]	0.70	11.87 ± 3.19	9.84 ± 3.42	−0.13	[−9.64, 9.38]	0.97
Urinary Phosphate (mg/24 h)	756.61 ± 295.95	679.79 ± 278.29	−76.81	[−192.1, 38.5]	0.19	760.33 ± 99.75	677.00 ± 233.77	−83.33	[−263.53, 96.87]	0.31
Urinary Calcium (mg/24 h)	**176.03 ± 83.94**	**242.25 ± 100.07**	**66.22**	**[29.24, 103.20]**	**0.009**	241.33 ± 96.60	226.00 ± 109.44	−15.33	[−125.33, 94.67]	0.75
TNF-Alfa (pg/mL)	1.82 ± 3.24	39.37 ± 163.09	37.55	[−8.25, 83.35]	0.10	2.09 ± 2.36	162.19 ± 356.56	−160.10	[−82.90, 403.10]	0.16
P3NP (μg/L)	10.75 ± 6.80	9.60 ± 5.67	−1.15	[−3.67, 1.37]	0.36	7.43 ± 2.16	8.76 ± 2.38	1.33	[−1.10, 3.76]	0.23
Sclerostin (pmol/L)	65.41 ± 54.79	66.85 ± 52.95	1.44	[−20.18, 23.06]	0.90	58.40 ± 46.91	96.36 ± 82.41	37.96	[−30.59, 106.51]	0.23
Leptin (ng/mL)	15.84 ± 13.26	19.22 ± 20.88	3.38	[−3.61, 10.37]	0.34	12.58 ± 5.18	15.06 ± 16.24	2.48	[−9.41, 14.37]	0.631
CTX (ng/mL)	0.292 ± 0.186	0.274 ± 0.242	−0.018	[−0.104, 0.068]	0.68	0.34 ± 0.04	0.34 ± 0.44	0.00	[−0.30, 0.30]	1.000
IRISIN (ng/mL)	198.72 ± 168.20	160.18 ± 119.30	−38.54	[−97.20, 20.12]	0.20	101.00 ± 101.06	139.18 ± 99.62	38.18	[−70.32, 146.68]	0.43
P1NP (ng/mL)	296.40 ± 163.22	313.65 ± 205.81	17.25	[−57.05, 91.55]	0.65	436.15 ± 160.12	342.91 ± 369.59	−93.24	[−378.34, 191.86]	0.46
EuroQoL-5D Index	0.936 ± 0.110	0.935 ± 0.103	−0.001	[−0.044, 0.042]	0.99	0.827 ± 0.000	0.937 ± 0.116	0.11	[0.03, 0.19]	0.013
EuroQoL-5D (VAS)	**74.85 ± 13.43**	**67.50 ± 16.49**	**−** **7.34**	**[** **−13.36, −1.34** **]**	**0.01**	**80.** **00 ± 0.00**	**60.00 ± 15.81**	**−20.00**	**[−30.80,−9.20]**	**0.003**
SPPB Total Score	10 (3–12)	9 (5–12)	0.622	[−0.591, 1.833]	0.552	8 (3–10)	8 (6–11)	−1.25	[−5.77, 3.27]	0.534
SPPB Balance	4 (1–4)	4 (1–4)	0.356	[−0.207, 0.919]	0.211	3 (1–4)	3.5 (1–4)	−0.25	[−2.39, 1.89]	0.790
SPPB Gait Speed	3 (1–4)	3 (1–4)	0.181	[−0.349, 0.712]	0.495	3 (1–3)	3 (1–4)	−0.55	[−2.40, 1.30]	0.505
SPPB Sit-to-Stand	3 (0–4)	3 (0–4)	0.000	[−0.589, 0.589]	1.000	1 (1–3)	2 (0–4)	−0.45	[−2.30, 1.40]	0.584
Handgrip Strength (kg)	24.59 ± 7.56	25.47 ± 9.82	0.88	[−2.64, 4.40]	0.62	**14.08 ± 1.95**	**18.80 ± 6.06**	**4.72**	**[0.28, 9.16]**	**0.04**
Gait speed (m/s)	0.805 ± 0.225	0.733 ± 0.266	0.07	[−0.171, 0.027]	0.15	0.787 ± 0.30	0.818 ± 0.17	0.03	[−0.25, 0.31]	0.80
ASMM (kg)	**17.16 ± 2.67**	**20.30 ± 5.22**	**3.14**	**[** **1.49, 4.7** **]**	**0.0003**	18.05 ± 1.60	21.07 ± 6.56	3.02	[−1.66, 7.70]	0.17
ASMM (kg)/h^2^ (m)	6.85 ± 1.05	7.16 ± 1.17	0.31	[−0.135, 0.755]	0.17	**6.04 ± 0.56**	**6.93 ± 0.34**	**0.88**	**[0.37, 1.41]**	**0.004**
IPAQ (MET)	3977.67 ± 4491.88	3935.29 ± 3408.62	−42.33	[−549.0, 464.2]	0.83	2324.063 ± 1396.6592	3301.364 ± 4300.8436	977.3	[−2183.1, 4137.7]	0.49
MET (total)	12,247.79 ± 2345.71	12,021.04 ± 3102.27	−226.75	[−1327.35, 873.85]	0.68	10,806.33 ± 2307.93	9729.80 ± 4996.79	1076.53	[−4989.23, 2836.17]	0.53
MET (week)	1791.61 ± 328.38	1870.38 ± 282.70	−78.76	[−44.33, 201.87]	0.22	1583.67 ± 331.54	1746.00 ± 178.16	162.33	[−140.37, 465.03]	0.24

Notes: Continuous variables are expressed as mean ± standard deviation or median with interquartile range; categorical variables are expressed as total number (%). Abbreviations: Body Mass Index (BMI), Alanine Aminotransferase (ALT), Aspartate Aminotransferase (AST), Total Alkaline Phosphatase (Total ALP), 25-Hydroxy Vitamin D (25OH-Vitamin D), Bone-specific Alkaline Phosphatase (bALP), Parathyroid Hormone (PTH), Tumor Necrosis Factor-alpha (TNF α), Procollagen Type III N-terminal Propeptide (P3NP), C-terminal Telopeptide of Type I Collagen (CTX), Procollagen Type I N-terminal Propeptide (P1NP) (µg/L), Cumulative Illness Rating Scale (CIRS), EuroQoL-5Dimension (EuroQoL-5D), Visual Analog Scale (VAS), Short Physical Performance Battery (SPPB), Handgrip Strength Examination (HGSE), Appendicular Skeletal Muscle Mass (ASMM), International Physical Activity Questionnaire (IPAQ), Metabolic Equivalent of Task (MET). Statistically significant results are reported in bold.

**Table 3:** Between-group comparison after 12 months of laboratory parameters, and functional outcomes in the general population and in osteosarcopenic subgroup, divided by type of water.

	**General Population**					**Osteosarcopenic Population**				
**Dependent Variable**	**J66 N = 48** **(M + SD)**	**SG9 N = 50 (M + SD)**	**Mean Difference**	**95% CI**	***p*** **-Value**	**J66 N = 8 (M + SD)**	**SG9 N = 12 (M + SD)**	**Mean Difference**	**95% CI**	***p*** **-Value**
BMI	24.63 ± 4.12	25.67 ± 3.82	1.04	[−0.55, 2.63]	0.20	22.10 ± 2.02	25.45 ± 4.34	3.35	[−0.06, 6.76]	0.05
Magnesium (mEq/L)	2.07 ± 0.13	2.07 ± 0.16	0.00	[−0.058, 0.058]	1.00	2.03 ± 0.07	2.009 ± 0.14	−0.021	[−0.133, 0.091]	0.67
Phosphate (mg/dL)	3.48 ± 0.42	3.58 ± 0.58	0.10	[−0.10, 0.30]	0.34	3.42 ± 0.46	3.62 ± 0.66	0.20	[−0.39, 0.79]	0.44
Calcium (mg/dL)	9.12 ± 0.27	9.10 ± 0.33	−0.080	[0.141, 0.101]	0.74	9.00 ± 0.19	8.88 ± 0.27	0.12	[−0.10, 0.34]	0.26
Total ALP (U/L)	68.42 ± 21.68	69.03 ± 16.67	0.61	[−7.17, 8.39]	0.88	70.40 ± 13.98	74.33 ± 13.23	−3.93	[−18.7, 10.9]	0.54
25OH-Vitamin D (ng/mL)	36.68 ± 11.55	36.08 ± 10.60	−0.60	[−5.05, 3.85]	0.79	46.34 ± 12.67	34.38 ± 11.95	−11.96	[−25.33, 1.41]	0.07
bALP (%)	9.98 ± 3.58	10.12 ± 3.50	0.14	[−1.28, 1.56]	0.84	10.32 ± 1.32	10.38 ± 3.21	0.06	[−2.39, 2.51]	0.95
PTH (pg/mL)	32.78 ± 11.57	29.44 ± 11.84	3.34	[−8.03, 1.35]	0.16	30.90 ± 14.25	29.88 ± 10.40	−1.02	[−14.87, 12.83]	0.87
Urinary Magnesium (mEq/24 h)	**9.03 ± 4.79**	**11.90 ± 5.93**	**2.87**	**[0.71, 5.03]**	**0.009**	**7.38 ± 3.31**	**11.82 ± 8.31**	**4.44**	**[** **−1.87, 10.75** **]**	**0.** **14**
Urinary Phosphate (mg/24 h)	599.15 ± 288.58	611.03 ± 228.96	−11.88	[−92.90, 116.7]	0.82	**385.20 ± 182.19**	**603.67 ± 156.71**	**218.47**	**[** **32.27, 404.67** **]**	**0.** **027**
Urinary Calcium (mg/24 h)	**160.82 ± 134.83**	**231.39 ± 113.46**	**−70.57**	**[20.52, 120.62]**	**0.006**	**85.80 ± 69.76**	**218.83 ± 125.65**	**133.03**	[29.33, 236.73]	**0.0** **18**
TNF-Alfa (pg/mL)	4.15 ± 3.26	18.77 ± 76.35	−14.62	[−6.81, 36.05]	0.18	3.30 ± 1.4	77.76 ± 178.45	74.46	[−47.34, 196.26]	0.19
P3NP (μg/L)	7.25 ± 2.75	7.31 ± 2.72	0.06	[−1.04, 1.16]	0.91	**5.97 ± 1.48**	**8.** **80 ± 1.99**	**2.83**	**[0.99, 4.67]**	**0** **.** **008**
Sclerostin (pmol/L)	84.81 ± 36.44	92.51 ± 46.28	7.70	[−8.97, 24.37]	0.36	76.80 ± 29.25	112.98 ± 92.45	36.18	[−31.52, 103.88]	0.24
Leptin (ng/mL)	14.05 ± 11.10	16.86 ± 17.37	2.81	[−3.01, 8.63]	0.34	10.49 ± 5.95	15.11 ± 8.98	4.62	[−3.28, 12.52]	0.21
CTX (ng/mL)	0.371 ± 0.205	0.322 ± 0.209	−0.049	[−0.132, 0.034]	0.24	0.41 ± 0.19	0.35 ± 0.21	−0.06	[−0.274, 0.154]	0.53
IRISIN (ng/mL)	53.41 ± 36.48	52.44 ± 26.24	−0.97	[−13.75, 11.81]	0.88	35.84 ± 13.75	48.47 ± 23.18	12.63	[−6.93, 32.19]	0.17
P1NP (ng/mL)	402.09 ± 223.45	385.39 ± 201.99	−16.70	[−102.2, 68.8]	0.70	365.60 ± 164.66	465.83 ± 310.48	100.23	[−152.17, 352.63]	0.38
EuroQoL-5D Index	0.91 ± 0.11	0.95 ± 0.09	0.037	[−0.0004, 0.0804]	0.05	0.85 ± 0.84	0.94 ± 0.09	0.09	[−0.615, 0.795]	0.77
EuroQoL-5D (VAS)	70.45 ± 15.33	69.09 ± 13.78	−1.36	[−7.22, 4.50]	0.65	76.00 ± 8.94	65.00 ± 12.25	−11	[−22.22, 0.22]	0.05
SPPB Total Score	10 (6–12)	9 (6–12)	0.171	−0.71 to 1.32	0.53	8 (6–11)	9 (7–12)	0.622	[−1.310, 2.554]	0.53
SPPB Balance	4 (1–4)	4 (1–4)	0.000	[3.577, 4.023]	1.00	4 (1–4)	4 (3–4)	0.000	[−0.303, 0.303]	1.00
SPPB Gait Speed	3 (1–4)	3 (1–4)	0.133	[2.676, 3.457]	0.56	3 (1–4)	3 (2–4)	0.133	[−0.325, 0.591]	0.56
SPPB Sit-to-Stand	3 (0–4)	3 (0–4)	0.066	[2.253, 3.147]	0.82	2 (1–3)	2 (0–4)	0.066	[−0.497, 0.629]	0.82
Handgrip Strength (kg)	22.28 ± 7.18	23.85 ± 8.98	1.57	[−1.69, 4.83]	0.34	17.3 ± 9.86	18.74 ± 6.49	1.44	[−7.92, 10.80]	0.73
Gait speed (m/s)	0.94 ± 0.30	0.85 ± 0.21	−0.09	[−0.194, 0.014]	0.09	0.78 ± 0.24	0.79 ± 0.15	0.01	[−0.215, 0.235]	0.92
ASMM (kg)	**17.61 ± 3.19**	**20.62 ± 5.68**	**3.01**	**[1.17, 4.85]**	**0.0016**	**16.35 ± 2.78**	**20.60** **± 4.26**	**4.25**	**[0.53, 7.97** **]**	**0.03**
ASMM (kg)/h^2^ (m)	**6.80 ± 0.99**	**7.79 ± 1.49**	**−0.99**	**[** **0.485, 1.495** **]**	**0.0002**	**6.39** **± 0.627**	**7.55 ± 0.69**	**1.16**	**[** **0.455, 1.865** **]**	**0.006**
IPAQ (MET)	3568.100 ± 4595.32	21,966.267 ± 98,552.99	18,398	[−9512, 46,318]	0.19	2425.00 ± 1537.425	1165.00 ± 612.88	−1260.000	[−2612.7, 92.7]	0.06
MET (total)	11,920.18 ± 2503.44	12,824.36 ± 2470.98	904	[−94, 1902]	0.07	10,426.0000 ± 2383.29709	12,027.50 ± 1810.45	1601.50	[−742.14, 3945.14]	0.15
MET (week)	1800.15 ± 389.12	1870.70 ± 343.38	70.55	[−76.8, 217.9]	0.34	1511.2000 ± 356.49923	1774.33 ± 276.51	263.13	[−89.65, 615.91]	0.12

Notes: Continuous variables are expressed as mean ± standard deviation or median with interquartile range; categorical variables are expressed as total number (%). Abbreviations: Body Mass Index (BMI), Alanine Aminotransferase (ALT), Aspartate Aminotransferase (AST), Total Alkaline Phosphatase (Total ALP), 25-Hydroxy Vitamin D (25OH-Vitamin D), Bone-specific Alkaline Phosphatase (bALP), Parathyroid Hormone (PTH), Tumor Necrosis Factor-alpha (TNF α), Procollagen Type III N-terminal Propeptide (P3NP), C-terminal Telopeptide of Type I Collagen (CTX), Procollagen Type I N-terminal Propeptide (P1NP) (µg/L), Cumulative Illness Rating Scale (CIRS), EuroQoL-5Dimension (EuroQoL-5D), Visual Analog Scale (VAS), Short Physical Performance Battery (SPPB), Handgrip Strength Examination (HGSE), Appendicular Skeletal Muscle Mass (ASMM), International Physical Activity Questionnaire (IPAQ), Metabolic Equivalent of Task (MET). Statistically significant results are reported in bold.

## Data Availability

The original contributions presented in this study are included in the article. Further inquiries can be directed to the corresponding author.

## References

[1] Vannucci L., Fossi C., Quattrini S., Guasti L., Pampaloni B., Gronchi G., Giusti F., Romagnoli C., Cianferotti L., Marcucci G. (2018). Calcium Intake in Bone Health: A Focus on Calcium-Rich Mineral Waters. Nutrients.

[2] Veldurthy V., Wei R., Oz L., Dhawan P., Jeon Y.H., Christakos S. (2016). Vitamin D, calcium homeostasis and aging. Bone Res..

[3] Goltzman D., Mannstadt M., Marcocci C. (2018). Physiology of the Calcium-Parathyroid Hormone-Vitamin D Axis. Vitamin D in Clinical Medicine.

[4] Fatima G., Dzupina A., BAlhmadi H., Magomedova A., Siddiqui Z., Mehdi A., Hadi N. (2024). Magnesium Matters: A Comprehensive Review of Its Vital Role in Health and Diseases. Cureus.

[5] Dominguez L.J., Mérida D.M., Donat-Vargas C., Banegas J.R., Veronese N., Barbagallo M., Rodríguez-Artalejo F., Guallar-Castillón P. (2025). Higher Magnesium Intake Is Associated with a Lower Risk of Frailty in Older Adults. J. Am. Med. Dir. Assoc..

[6] Pietropaolo G., Castiglioni S., Maier J.A., Wolf F.I., Trapani V. (2025). Magnesium Preserves Calcium Homeostasis and Contributes to Protect Myotubes from Inflammation-Induced Damage. Int. J. Mol. Sci..

[7] Kanis J.A., McCloskey E.V., Johansson H., Oden A., Melton L.J., Khaltaev N. (2008). A reference standard for the description of osteoporosis. Bone.

[8] Cruz-Jentoft A.J., Bahat G., Bauer J., Boirie Y., Bruyère O., Cederholm T., Cooper C., Landi F., Rolland Y., Sayer A.A. (2019). Sarcopenia: Revised European consensus on definition and diagnosis. Age Ageing.

[9] Clynes M.A., Gregson C.L., Bruyère O., Cooper C., Dennison E.M. (2021). Osteosarcopenia: Where osteoporosis and sarcopenia collide. Rheumatology.

[10] Tang B.M., Eslick G.D., Nowson C., Smith C., Bensoussan A. (2007). Use of calcium or calcium in combination with vitamin D supplementation to prevent fractures and bone loss in people aged 50 years and older: A meta-analysis. Lancet.

[11] Bolland M.J., Leung W., Tai V., Bastin S., Gamble G.D., Grey A., Reid I.R. (2015). Calcium intake and risk of fracture: Systematic review. BMJ.

[12] Thabit H., Barry M., Sreenan S., Smith D. (2011). Proximal myopathy in lacto-vegetarian Asian patients responding to Vitamin D and calcium supplement therapy—Two case reports and review of the literature. J. Med. Case Rep..

[13] Hodges J.K., Cao S., Cladis D.P., Weaver C.M. (2019). Lactose Intolerance and Bone Health: The Challenge of Ensuring Adequate Calcium Intake. Nutrients.

[14] Moretti A., Liguori S., Paoletta M., Migliaccio S., Toro G., Gimigliano F., Iolascon G. (2023). Bone fragility during the COVID-19 pandemic: The role of macro- and micronutrients. Ther. Adv. Musculoskelet. Dis..

[15] Matković V., Kostial K., Simonović I., Buzina R., Brodarec A., Nordin B.E. (1979). Bone status and fracture rates in two regions of Yugoslavia. Am. J. Clin. Nutr..

[16] Cepollaro C., Orlandi G., Gonnelli S., Ferrucci G., Arditti J.C., Borracelli D., Toti E., Gennari C. (1996). Effect of calcium supplementation as a high-calcium mineral water on bone loss in early postmenopausal women. Calcif. Tissue Int..

[17] Hopewell S., Chan A.W., Collins G.S., Hróbjartsson A., Moher D., Schulz K.F., Tunn R., Aggarwal R., Berkwits M., Berlin J.A. (2025). CONSORT 2025 statement: Updated guideline for reporting randomised trials. BMJ.

[18] Iolascon G., de Sire A., Calafiore D., Benedetti M.G., Cisari C., Letizia Mauro G., Migliaccio S., Nuti R., Resmini G., Gonnelli S. (2020). Multifactorial Assessment of Risk of Falling in 753 Post-Menopausal Women: A Multicenter Cross-Sectional Study by the Italian Group for the Study of Metabolic Bone Diseases. Clin. Interv. Aging.

[19] Asano K., Kabasawa K., Takachi R., Sawada N., Tsugane S., Ito Y., Narita I., Nakamura K., Tanaka J. (2025). Association of dietary calcium intake with risk of falls in community-dwelling middle-aged and older adults. J. Nutr. Health Aging.

[20] Liguori S., Moretti A., Paoletta M., Gimigliano F., Iolascon G. (2024). Role of Magnesium in Skeletal Muscle Health and Neuromuscular Diseases: A Scoping Review. Int. J. Mol. Sci..

[21] Moretti A. (2021). What is the role of magnesium for skeletal muscle cramps? A Cochrane Review summary with commentary. J. Musculoskelet. Neuronal Interact..

[22] Schneider I., Greupner T., Hahn A. (2017). Magnesium bioavailability from mineral waters with different mineralization levels in comparison to bread and a supplement. Food Nutr. Res..

[23] Canfora I., Tarantino N., Pierno S. (2022). Metabolic Pathways and Ion Channels Involved in Skeletal Muscle Atrophy: A Starting Point for Potential Therapeutic Strategies. Cells.

[24] Polito A., Barnaba L., Ciarapica D., Azzini E. (2022). Osteosarcopenia: A Narrative Review on Clinical Studies. Int. J. Mol. Sci..

[25] Romagnoli C., Brandi M.L. (2021). Muscle Physiopathology in Parathyroid Hormone Disorders. Front. Med..

[26] Chien M.Y., Huang T.Y., Wu Y.T. (2008). Prevalence of sarcopenia estimated using a bioelectrical impedance analysis prediction equation in community-dwelling elderly people in Taiwan. J. Am. Geriatr. Soc..

[27] Zhang J., Zhang N., Lu J., Liu S., Lin Y., Ma G. (2025). Seasonal fluctuation of total water intake and hydration status among young men and women: A prospective cohort study. Front. Nutr..

